# A black macule on the male nipple

**DOI:** 10.1016/j.jdcr.2025.11.015

**Published:** 2025-11-20

**Authors:** Noriyoshi Fujii, Akemi Fukuda, Nozomi Kawahara, Keiko Kobayashi

**Affiliations:** a Department of Dermatology, Mito Red Cross Hospital, Mito, Ibaraki, Japan; b Department of Dermatology, Institute of Medicine, University of Tsukuba, Tsukuba, Ibaraki, Japan

**Keywords:** blue-gray globules, male breast cancer, melanocyte colonization, pigmented macule

## Case presentation

**Fig 1:**
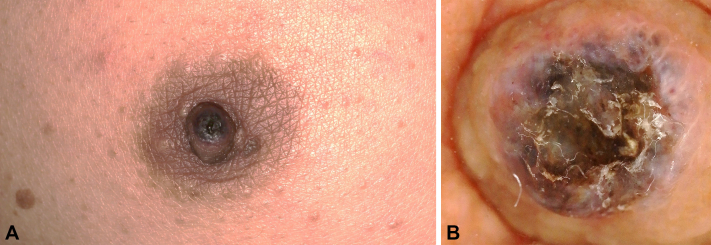
**A,** 5 × 5 mm black macule with thin crust on the right nipple–areola complex. **B,** Dermoscopy: blue-gray globules and irregular *dark dots* with central surface erosion/ulceration; no clear arborizing vessels.

A 55-year-old man with no significant medical history presented with a 2-month history of a pigmented macule and intermittent scaling on the right nipple–areola complex (Fig 1, *A*). He denied pain, discharge, bleeding, fever, weight loss, and trauma. Examination revealed a well-demarcated 5 × 5 mm dark macule with a thin adherent crust; no subareolar mass or axillary lymphadenopathy was detected. Dermoscopy showed blue-gray globules and irregular dark dots with central surface erosion/ulceration; arborizing vessels were not clearly identified (Fig 1, *B*). Baseline laboratory tests (including carcinoembryonic antigen and cancer antigen 15-3) and axillary ultrasonography were unremarkable. A complete excisional biopsy was performed in a circular manner with 2-mm clinical margins, including full-thickness dermis to the superficial subcutis.

**Fig 2:**
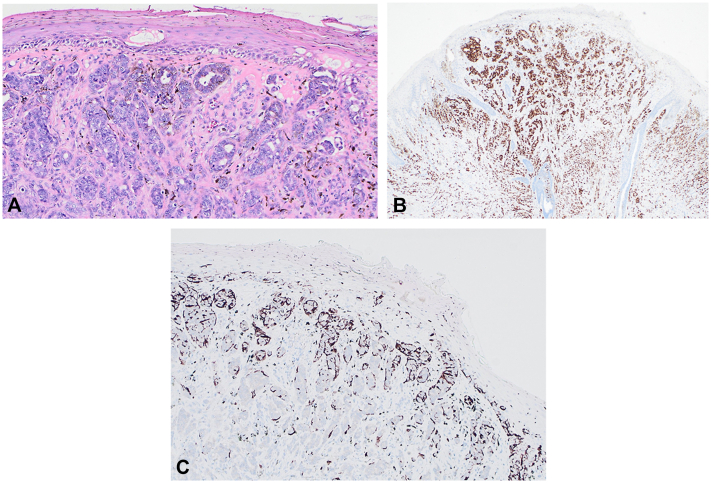
**A,** H&E: superficial crust over dermal nests/cords; dendritic melanocytes and melanophages. **B,** ER immunohistochemistry: strong nuclear staining. **C,** Melan-A immunohistochemistry: dendritic melanocytes around nests. *ER*, Estrogen receptor; *H&E*, hematoxylin and eosin.

Histopathology shows a superficial crust with focal basal vacuolar alteration and, beneath it, a dermal cellular proliferation with nested/cord-like architecture, accompanied by dendritic melanocytes and melanin-laden melanophages (Fig 2, *A*). Immunostains for estrogen receptor (Fig 2, *B*) and Melan-A (Fig 2, *C*) are provided for clinicopathologic correlation.

**Question: What is the most likely diagnosis for this patient?**

**A.** Pigmented nodular basal cell carcinoma
**B.** Pigmented eccrine porocarcinoma
**C.** Nodular malignant melanoma
**D.** Invasive ductal carcinoma
**E.** Pigmented mammary Paget disease

## Answer discussion

The correct answer is **D.**

Male breast cancer is an uncommon entity, representing approximately 1% of all breast malignancies.^1^ It most frequently arises in the central retroareolar region, often involving the nipple.^1^ Clinically, a painless mass is the typical presentation; pigmented epidermal change is exceedingly rare, and overall management generally mirrors that in women.^1^ When pigmentation is present, bedside impression may resemble melanoma or basal cell carcinoma, and reported male cases describe deceptive dermoscopic patterns—including blue-gray areas, blue-white structures, and occasionally corkscrew vessels—supporting biopsy of even small pigmented nipple lesions.^2^^,^^3^

The patient in this case had a small, sharply demarcated black macule with blue-gray structures and central surface erosion on dermoscopy, features that can confound clinical judgment. The lesion was excised circularly with a narrow 2-mm margin to secure diagnostic accuracy, consistent with melanoma biopsy recommendations, while minimizing cosmetic deformity of the nipple–areola complex. Histopathology revealed a predominantly dermal tubule-forming epithelial neoplasm with minimal epidermal involvement, absence of Paget cells, or pagetoid spread, leading to a diagnosis of invasive ductal carcinoma. Immunohistochemically, tumor nuclei demonstrated strong estrogen receptor positivity. Notably, Melan-A failed to stain the neoplastic cells, instead highlighting dendritic melanocytes at the periphery of carcinoma nests. These findings support a diagnosis of a nonmelanocytic malignancy with secondary pigmentation, as opposed to primary melanoma or pigmented mammary Paget disease.

The most plausible basis for pigmentation is melanocyte colonization: disruption of the dermoepidermal junction permits epidermal melanocytes to migrate into and around carcinoma nests and transfer melanin via dendritic processes to adjacent tumor cells, producing intracellular melanin.^4^ Paracrine signaling is also likely to contribute, as breast carcinoma can express basic fibroblast growth factor, which promotes melanocyte migration and proliferation.^5^ In male patients with a pigmented nipple macule, integration of dermoscopy, histopathology, and immunohistochemistry is indispensable to prevent diagnostic errors and to facilitate timely definitive therapy.

## Conflicts of interest

None disclosed.
